# Genetic Polymorphisms and Diversity in Nonalcoholic Fatty Liver Disease (NAFLD): A Mini Review

**DOI:** 10.3390/biomedicines11010106

**Published:** 2022-12-30

**Authors:** Siti Aishah Sulaiman, Vicneswarry Dorairaj, Muhammad Nafiz Haidi Adrus

**Affiliations:** UKM Medical Molecular Biology Institute (UMBI), Universiti Kebangsaan Malaysia (UKM), Jalan Yaa’cob Latiff, Cheras, Kuala Lumpur 56000, Malaysia; p105311@siswa.ukm.edu.my (V.D.); p121290@siswa.ukm.edu.my (M.N.H.A.)

**Keywords:** NAFLD, genetics, diversity, ethnic difference, polymorphism, steatosis

## Abstract

Nonalcoholic fatty liver disease (NAFLD) is a common liver disease with a wide spectrum of liver conditions ranging from hepatic steatosis to nonalcoholic steatohepatitis (NASH), fibrosis, cirrhosis, and hepatocellular carcinoma. The prevalence of NAFLD varies across populations, and different ethnicities have specific risks for the disease. NAFLD is a multi-factorial disease where the genetics, metabolic, and environmental factors interplay and modulate the disease’s development and progression. Several genetic polymorphisms have been identified and are associated with the disease risk. This mini-review discussed the NAFLD’s genetic polymorphisms and focusing on the differences in the findings between the populations (diversity), including of those reports that did not show any significant association. The challenges of genetic diversity are also summarized. Understanding the genetic contribution of NAFLD will allow for better diagnosis and management explicitly tailored for the various populations.

## 1. Introduction

Nonalcoholic fatty liver disease (NAFLD) is a spectrum of liver disease that ranges from simple steatosis to nonalcoholic steatohepatitis (NASH), fibrosis, and ultimately cirrhosis and hepatocellular carcinoma (HCC). Previous studies suggest that NAFLD is strongly associated with other metabolic disorders, including metabolic syndrome, obesity, insulin resistance (IR), and type 2 diabetes (T2D) [1,2]. Currently, NAFLD is the most common liver disease affecting 30% of the global population, with a higher prevalence in Asia, Latin America, and Middle East-North Africa [3,4]. However, specific ethnicities are more protected against NAFLD than others [5,6], whereas some individuals having the same genetic polymorphisms would increase their predisposition to NAFLD [5,7].

Genetic and environmental factors modulate the risk of NAFLD disease development and its progression. Several genetic polymorphisms associated with NAFLD risk have been published before by genome-wide association studies (GWAS) and single or multiple-gene studies [8,9,10]. These genetic polymorphisms could implicate the pathways involved in NAFLD, such as IR, fatty acid metabolism, oxidative stress, and inflammation. Thus, this mini-review discussed the NAFLD’s genetic polymorphisms focusing on the differences between the populations (diversity), including no significant association. The challenges of genetic diversity are also addressed.

## 2. NAFLD Pathogenesis and Progression

Understanding the pathogenesis of NAFLD disease is vital in determining the genetics’ role in disease development and progression. NAFLD disease development is widely agreed to be a “multiple hits” theory, assuming the complex interplay of metabolic, genetic and epigenetics, and environmental factors [11,12,13].

One of the critical processes in NAFLD disease development is the dysregulation of the lipid metabolism that drives hepatic lipid accumulation or steatosis. The majority of the hepatic lipids or triglycerides (TG) content comes from the adipocytes-derived circulating free fatty acids (FFAs), which are mediated by the lipase enzymes [14,15]. One such enzyme is the adipose triglyceride lipase (ATGL), encoded by the Patatin-like phospholipase domain containing 2 (*PNPLA2*) gene. ATGL enzyme initiates the TG breakdown by hydrolyzing the ester bond into producing the diacylglycerol. This step leads to the downstream cascade of lipid breakdown by recruiting other lipases to produce the end products of glycerol and FFAs [16]. In the presence of IR, uncontrolled lipolysis could cause a significant rise in circulating FFAs [17]. Therefore, genetic modifications in these lipid metabolism regulators could contribute to NAFLD development.

Among the patients with steatosis, about 25% of them will have inflammation, hepatocyte ballooning, and cell death (NASH) [18]. Although various hypotheses are reported to explain this NASH progression, the actual molecular mechanism is partly understood. Excess lipids trigger oxidative stress, which, in turn, dramatically reduces the ability of FFAs removal by the mitochondrial β-oxidation and induces endoplasmic reticulum stress [19]. Reactive oxygen species (ROS) suppresses the expression of peroxisome proliferator-activated receptor-α (*PPARA*), an essential transcriptional factor of FFA oxidation [20,21], thus contributing to further lipid dysregulation. In another aspect, ROS also interact with unsaturated FFAs to enable lipid peroxidation and produce the Malondialdehyde, a marker of oxidative damage [22]. Therefore, ROS-mediated mitochondrial dysfunction and lipid dysregulation could compromise FFAs removal and oxidative phosphorylation, thus initiating a continuous cycle of mitochondrial dysfunction.

In addition to mitochondrial dysfunction, aberrant cytokine and inflammatory factors are evident in NASH and fibrosis progression. Hepatic resident-macrophage (Kupffer cells) play a vital role in liver inflammation. Activated Kupffer cells produce chemokines and cytokines such as Interleukin 6 (IL-6), Tumor necrosis factor-α (TNF-α), and C-C-motif chemokine ligand-2 (CCL2) that further magnify the effects of IR and inflammation thus triggering the hepatocyte injury and fibrosis [23]. The activation of M1 Kupffer cells (proinflammatory type) contributes to fibrosis pathogenesis, whereas the activation of M2 Kupffer cells (anti-inflammatory type) protects such progression [24,25]. Furthermore, the animal model of high-fat diet mice showed that the ratio of M1 to M2 Kupffer cells leads to different outcomes, as mice with a higher M2:M1 ratio are less likely to develop liver lesions, and vice versa for the mice with a high M1:M2 ratio [26]. Damaged hepatocytes and activated Kupffer cells release various proinflammatory cytokines and fibrotic inducers such as TNF-α, platelet-derived growth factor, CCL3, CCL5, and transforming growth factor-β, which, in turn, activates HSCs proliferation [23,27]. This HSCs activation increases expressions of α-smooth muscle actin, forming the stress fibers and depositing extracellular matrix (ECM) components [28]. Previous studies showed that genetic alterations or inhibition of the chemokines and their receptors had improved NASH in mice [29,30,31,32,33], indicating the possible role of genetic modulation in NASH and fibrosis progression.

## 3. Genetic Polymorphisms of NAFLD

The first reported GWAS study of NAFLD patients was in 2008 in a multi-cohort of Hispanics, African Americans, and European Americans [34]. In this study, the genetic variant of Patatin-like phospholipase domain-containing 3 (*PNPLA3*), rs738409, also known as I148M, was associated with greater lipid contents, even after adjustments for the ethnicity, body mass index (BMI), diabetes status, and alcohol intake [34]. After that, several studies also reported the effects of the I148M variant and other variants associated with NAFLD in different populations (Table 1).

### 3.1. PNPLA3 Loci

**Figure 1:**
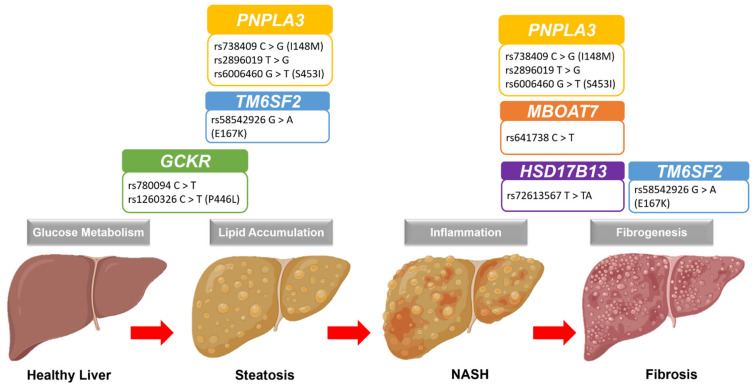
Graphical representation of the NAFLD progression and the most common genetic polymorphisms. Abbreviation: GCKR: glucokinase regulatory protein; HSD17B13: hydroxysteroid 17β-dehydrogenase; MBOAT7: membrane-bound O-acyltransferase domain-containing 7; NAFLD: nonalcoholic fatty liver disease; NASH: nonalcoholic steatohepatitis; PNPLA3: patatin-like phospholipase domain-containing 3; TM6SF2: transmembrane 6 superfamily member 2.

The gene *PNPLA3* produces a triacylglycerol lipase (adiponutrin) responsible for triacylglycerol hydrolysis. The presence of the I148M variant reduces this lipase enzymatic activity and promotes hepatic steatosis (Figure 1) [116]. A recent meta-analysis of the I148M variant on NAFLD risk showed that individuals carrying the minor G-allele had a 19% higher risk of developing NAFLD, and the risk increased to 105% for individuals with both GG alleles [117]. Notably, this effect of the I148M variant was independent of IR and lipid levels [118], though diets may interact with the effects. A study of Hispanic children with the I148M variant showed high hepatic lipid contents when consuming carbohydrate-rich diets [119]. High levels of carbohydrates facilitate lipid metabolism via the activation of transcription factor sterol regulatory binding protein-1c that regulates the *PNPLA3* expression [120,121]. Therefore, the effect of the I148M variant may be higher for those individuals that consume high carbohydrate-rich diets, suggesting the gene-environment interplay.

A similar effect of the I148M variant is evident for more advanced stages of NAFLD. In a meta-analysis of 16 studies, the homozygous GG alleles confer a 3.5-fold greater risk of having NASH and a 3.2-fold higher risk of having fibrosis [122]. In a more recent meta-analysis of 13,817 individuals with NAFLD, this I148M variant confers a 2.54-fold greater risk of having NASH [123], with a significant dose-dependent of the G allele [117,123]. A similar association was also observed between the I148M variant and progression to cirrhosis, in which the presence of one G allele confers a 2-fold higher risk of cirrhosis, and homozygous GG alleles confer 3-fold higher risk compared to CC genotypes [51]. Thus, the *PNPLA3* I148M variant has been incorporated into various predictive models to diagnose NAFLD disease severity and progression [115,124,125,126].

### 3.2. TM6SF2 Loci

Another reported SNP in NAFLD is the rs58542926 (E167K) variant from the Transmembrane 6 superfamily, member 2 (*TM6SF2*) gene. This E167K variant was associated with a higher risk of having NAFLD, hepatic steatosis, and advanced fibrosis but not inflammation (Figure 1) [127,128,129]. Moreover, this effect of the E167K variant is more significant in children than adults [128]. Although the effect of the E167K variant is lower compared to *PNPLA3* I148M, the individuals carrying both I148M and E167K variants had a double or additive risk of having NAFLD [86], indicating the gene–gene interplay in the disease. There are also leaner NAFLD individuals carrying the E167K variant than obese or overweight individuals [130], thus highlighting its specific role in developing NAFLD. Notably, most previous studies of “lean” NAFLD are from Asia [131], and this is consistent with the high frequency of the E167K variant in East Asians. Across the populations, the minor T allele frequency is more common in East Asians (~34%) than in Europeans (~26%), Hispanics (~10%), and Africans (~6%) [132]. However, there is a lack of genetic diversity to conclude this relationship due to the underrepresentation of other populations.

Although it is debatable, there is evidence that NAFLD individuals with the E167K variant have a low risk of having coronary artery disease (CAD) [88,133,134]. TM6SF2 is an ER transmembrane protein predominantly found in the liver, kidney, and small intestine cells and responsible for regulating the secretion of lipoproteins [135]. The E167K variant causes a loss of this protein function and consequently reduces the secretion of very low-density lipoprotein (VLDL) [136]. This protective effect of the E167K variant was further replicated in a large meta-analysis GWAS study of 60,801 CAD individuals [137] and an exome study of more than 300,000 individuals [138]. This dual conflicting effect of the E167K variant on NAFLD and CAD risks adds more to the complex pathophysiology of NAFLD and its related cardiometabolic risk.

### 3.3. GCKR Loci

The *GCKR* gene encodes a glucokinase regulator by forming a complex with glucokinase and diverts its location to the nucleus, affecting hepatic glucose storage and metabolism (Figure 1) [139]. Inhibition of glucokinase activity is one of the mechanisms for controlling the rate of hepatic glucose metabolism and lipogenesis [140]. Thus, any variant that affects the functionality of GCKR protein may contribute to the NAFLD risk. One of the most reported *GCKR* variants is the rs780094, and the minor T-allele increases the risk of having NAFLD and hepatic steatosis [141,142]. Interestingly, other studies showed that the rs780094 variant was protective against T2D risk [143]. Consistent with the role of GCKR in glucose metabolism, this variant’s presence could increase the glucokinase rate due to a lack of inhibition from the GCRK protein. This lack of GCRK inhibition is further evident in the studies investigating the effect of another *GCKR* rs1260326 (P446L) variant. The loss of GCKR function due to the P446L variant was associated with lower fasting glucose and insulin levels but higher hepatic lipid content [50,88,144]. Uncontrolled glucokinase activity causes a high malonyl-CoA (product of glucose metabolism) level blocking the FFA oxidation via the inhibition of carnitine-palmytoyltransferase-1, and malonyl-CoA also promotes lipogenesis by becoming its substrate, thus increasing lipid accumulation [140]. Moreover, both *GCKR*-P446L and *PNPLA3*-I148M variants have synergistic effects on the NAFLD risk, particularly on hepatic steatosis. A study of children and adolescents with obesity showed that both variants were independently associated with hepatic lipid contents. The combined analysis of both variants’ effects explained the hepatic lipid content variability in different ethnicities (39% in African Americans, 32% in Caucasians, and 15% in Hispanics) [145], indicating the gene–gene interplay for lipid regulation in the youth.

### 3.4. MBOAT7 Loci

Another reported SNP in NAFLD is rs641738 from the membrane-bound O-acyltransferase domain-containing 7 (*MBOAT7*) gene. Individuals carrying the minor T-allele have reduced expression of *MBOAT7* and hepatic inflammation and fibrosis (Figure 1) [146,147]. *MBOAT7* gene encodes the lysophosphatidylinositol acyltransferase enzyme that adds the arachidonic acid (AA) into the membrane phospholipids, phosphatidylinositol (PI) [148], and a deficiency of MBOAT7 protein leads to hepatic inflammation-mediated fibrosis [149]. Consistent with this, T-allele in rs641738 was associated with liver fibrosis in NAFLD individuals [47,103,104].

### 3.5. HSD17B13 Loci

Among the SNPs associated with NAFLD, one splicing site SNP rs72613567 in hydroxysteroid 17β-dehydrogenase (*HSD17B13*) gene confers a protective effect on NAFLD risk, particularly lowering the inflammation, NASH, and fibrosis (Figure 1) [150]. The insertion of TA nucleotides causes an early termination and produces a truncated protein of hepatic lipid-droplet protein HSD17B13, resulting in a loss of this protein function [111]. Although the information about this protein is limited, individuals carrying the rs72613567 variant have a lower risk of NASH and fibrosis, though no effect was observed on lipid levels or hepatic steatosis [109,110,111,112,113,115]. Interestingly, this protective effect of the rs72613567 variant is evident even in individuals carrying the *PNPLA3* I148M variant by lowering fibrosis risk [111,114]. However, a recent study reported that the protective effects of the rs72613567 variant could be limited to specific groups of individuals that are either women or individuals aged more than 45 years or having diabetes or obesity and individuals with the *PNPLA3* I148M variant [110], though these findings need further clarifications.

### 3.6. Other Genetic Loci

There are also rare variants associated with NAFLD. Most of these SNPs have been explicitly identified for a single population or have only been reported by a few studies (Table 2).

#### 3.6.1. Loci in Energy Metabolism

One NAFLD variant involved in energy metabolism is the rs4240624 variant near the protein phosphatase-1 regulatory subunit-3B (*PPP1R3B*) gene. This noncoding rs4240624 variant was associated with hepatic steatosis, higher cholesterol, and lower fasting glucose [39]. Further study also showed that the PPP1R3B protein promotes glycogen storage in the liver via activating glycogen synthase and inhibiting glycogen breakdown [151], suggesting the role of this gene in the development of NAFLD. Another SNP of *PPP1R3B*, rs61756425, was associated with NAFLD disease severity [49], though more validation is needed to confirm this relationship.

Another reported SNP is the rs8192678 (G482S) from the PPARG coactivator-1 alpha (*PPARGC1A*) gene. This G482S variant was associated with a higher risk of having NAFLD in Iranians, and Chinese Han populations, though no association was observed in the lipid or glucose profile [152,153]. A recent study in the Chinese Hans population showed that this G482S was also associated with NASH [154], and this finding was replicated in the Japanese population but with another *PPARGC1A* SNP of rs2290602. Although the effects of *PPARGC1A* SNPs are only reported in these three populations, the polymorphisms of *PPARGC1A* have been linked to many other metabolic diseases [155], thus highlighting its possible contribution to NAFLD disease.

**Table 2:** Summary of the rarely reported single nucleotide polymorphism (SNP) associated with NAFLD.

Gene	SNP	Population Study	SNP Effects	Reference(s)
*PPP1R3B*	rs4240624 G > A/C	Amish, Family Heart Study, and Framingham Heart Study	Associated with NAFLD risk	[39]
		Caucasian, African, and Mexican Americans	Associated with steatosis	[92]
	rs61756425 G > T	Italian	Associated with NAFLD severity	[49]
*PPARGC1A*	rs8192678 G > A (G482S)	Iranian, Chinese	Associated with NAFLD and NASH risk	[152,153,154]
	rs2290602 G > T	Japanese	Associated with NAFLD and NASH risk	[156]
*SAMM50*	rs3761472 A > G	Chinese, Korean, and Japanese	Associated with NAFLD risk	[48,157,158,159]
		Indian	Associated with NASH	[72]
	rs738491 C > T	Chinese, Japanese	Associated with NAFLD risk	[48,157]
	rs2143571 G > A	Chinese, Korean, Japanese	Associated with NAFLD risk	[48,157,159]
*APOC3*	rs2070666 T > A	Chinese	Associated with hepatic lipid content	[160]
	rs2854116 (T-455C)	European	No association with NAFLD risk	[161]
		Indian	Associated with NAFLD and lipid content	[162,163]
	rs2854117 (C-482T)	European	No association with NAFLD risk	[161]
		Indian	Associated with NAFLD and lipid content	[162,163]
*ATGR1*	rs2276736 A > G/T rs3772630 T > A/C rs3772627 A > G	Malaysians (Malays, Chinese, and Indians)	Associated with NAFLD risk in the presence of *PNPLA3* I148M variant	[164]
		Japanese	Associated with NAFLD risk and fibrosis	[165]
*GATAD2A*	rs4808199 G > A	Japanese	Associated with NAFLD	[74]
*IL27*	rs4788084 C > T	Indian	Associated with hepatic lipid content	[72]
*LPIN1*	rs13412852 C > T	Italian	Tended to associate with lower NASH	[166]
*LYPLAL1*	rs12137855 C > T	Amish, Family Heart Study, Framingham Heart Study, Finnish	Associated with NAFLD risk and steatosis	[39,101]
*PEMT*	rs7946 C > T (V175M)	Chinese, Caucasian	Associated with NAFLD risk	[167,168,169]
		Indian	Associated with NAFLD risk in lean individuals	[170]
		Korean	No association with NAFLD risk	[171]
*MTTP*	-493C/G	Japanese	Associated with NASH	[172]
		Italian, Brazilian	No association with NAFLD risk	[173,174,175]
*PARVB*	rs5764455 A > C/G	Japanese	Associated with NASH	[48]
	rs2073080 C > A/T	Indian, Finnish	Associated with steatosis	[66,101]
*SOD*	rs4880 A > G (C47T)	Italian and European	Associated with hepatic steatosis and fibrosis	[176]
		Japanese	Associated with NASH	[172]
*REPIN1*	12 bp deletion	Germany	Associated with lower NAFLD severity	[177]
*UCP3*	rs1800849 C > T (-55CT)	Spaniards	Associated with NASH	[178]

Abbreviation: AGTR1: angiotensin II receptor type 1; APOC3: apolipoprotein C3; GATAD2A: GATA zinc finger domain containing 2A; IL27: interleukin 27; LPIN1: lipin-1; LYPLAL1: lysophospholipase-like 1; MTTP: microsomal triglyceride transfer protein; NAFLD: nonalcoholic fatty liver disease; NASH: nonalcoholic steatohepatitis; REPIN1: replication initiator 1; PARVB: parvin beta; PEMT: phosphatidylethanolamine N-methyltransferase; PPARGC1A: PPARG coactivator 1 alpha; PPP1R3B: protein phosphatase-1 regulatory subunit 3B; SAMM50: SAMM50 sorting and assembly machinery component; SOD2: superoxide dismutase 2; UCP3: uncoupling protein 3.

#### 3.6.2. Loci in Mitochondrial Regulation

The SAMM50 sorting and assembly machinery component (*SAMM50*) gene polymorphisms also contribute to NAFLD risk. Three SNPs, rs3761472, rs738491, and rs2143571, were associated with a greater risk of having NAFLD in East Asians, including Chinese Han, Korean, and Japanese populations [48,157,158,159]. This finding was replicated again in the Indian population with a significant association with NASH, though it is for one SNP only (rs3761472) [72]. The exact mechanism of how polymorphism in the *SAMM50* gene could lead to NAFLD is partly understood. Since SAMM50 is responsible for maintaining the mitochondrial structure and assembly of respiratory chain complexes, any modifications on the protein will lead to mitochondrial dysfunction and FFA oxidation [158], known processes in NAFLD disease development. In addition to SAMM50, other oxidative stress-associated SNPs, such as rs4880 (C47T) in superoxide dismutase 2 (*SOD2*) [172,176] and rs1800849 (-55CT) in uncoupling protein 3 (*UCP3*) [178] genes, also associated with NAFLD, particularly with NASH. Despite limited findings on the oxidative stress-related SNPs in NAFLD, the role of cellular oxidative stress is well explored in the development of NAFLD and NASH.

#### 3.6.3. Loci in Cholesterol Polymorphism

The cholesterol gene polymorphism also contributes to NAFLD risk. The SNPs in apolipoprotein C3 (*APOC3*) rs2070666, rs2854116, and rs2854117 were associated with NAFLD risk [160,162,163]. This *APOC3* gene encodes a component of VLDL (apolipoprotein C3) that inhibits lipoprotein and hepatic lipase, and loss of APOC3 protein function was associated with high triglycerides [179]. Another cholesterol related-gene is microsomal triglyceride transfer protein (*MTTP*), and its -493C/G intronic variant was reported to increase NASH risk in the Japanese population [172]. The heterodimeric MTTP protein is responsible for transporting lipid molecules, including triglycerides and cholesterol ester, and apolipoprotein B assembly. Therefore, any modification of the *MTTP* gene will lead to the absence of VLDL, known as abetalipoproteinemia [180] and, hence, more significant hepatic lipid accumulation. Previous studies also identified other NAFLD variants such as the Lipin-1 (*LPIN1*) rs13412852 [166], interleukin 27 (*IL27*) rs4788084 [72], GATA zinc finger domain containing 2A (*GATAD2A*) rs4808199 [74], lysophospholipase-like 1 (*LYPLAL1*) rs12137855 [39,101], angiotensin II receptor type 1 (*ATGR1*) rs2276736, rs3772630, and rs3772627, phosphatidylethanolamine N-methyltransferase (*PEMT*) rs7946 V175M [167,168,169,170,171], replication initiator 1 (*REPIN1*) 12-bp deletion [177], and parvin beta (*PARVB*) rs5764455 [48] and rs2073080 [66,101].

#### 3.6.4. Copy Number of Variants (CNV)

Another genetic change associated with NAFLD is the copy number variants (CNV). Currently, five published papers reported CNV association with NAFLD risk. The first study used the GWAS analysis of CNV in 10 Malaysian individuals with simple steatosis and 39 individuals with NASH [181]. In this study, the most common CNV is the 14q11.2 region, which is present in 53.8% of NASH individuals, and within this region, there are a group of olfactory receptor (OR) family genes. Two novel CNVs were also identified, the 13q12.11 and 12q13.2 regions, consisting of the exportin 4 (*XPO4*) and phosphodiesterase 1B (*PDE1B*) genes, respectively, and these CNVs were associated with NASH progression [181]. Following that, a larger study of Malaysian NAFLD individuals confirmed that the duplication of 13q12.11 (*XPO4* gene) was associated with NASH risk. The gain of an additional copy of 13q12.11 was associated with higher serum triglycerides and ALT enzyme levels [182]. The involvement of *XPO4* CNV in NAFLD was replicated in another study of Caucasians with metabolic-NAFLD (MAFLD), and *XPO4* CNV was associated with fibrosis [183]. Therefore, these findings confirm the possible involvement of *XPO4* CNV in modulating the risk for NAFLD and fibrosis.

Other studies also reported different CNVs associated with NAFLD risk. A more recent NAFLD study of the Chinese population showed that 338 autosomal CNVs were identified for NAFLD, and the deletion of NLR Family Pyrin Domain Containing 4 (*NLRP4*) CNV was associated with NAFLD risk [184]. Another study of Chinese NAFLD individuals reported that the deletion of carboxylesterase 1 (*CES1*) CNV was associated with NAFLD risk (OR: 2.75) [185]. Although the evidence for CNV on NAFLD risk is limited, the replication of *XPO4* CNV association in two different populations may indicate that the CNVs could modulate the NAFLD risk. Thus, more future study is needed to determine this relationship.

From these findings, most of the NAFLD genetic polymorphisms are within the genes involved in regulating hepatic lipid metabolism, and the genetic contribution to NAFLD risk is considered significant. Therefore, these genetic variants are potential diagnostic tools to screen susceptible individuals for NAFLD. However, some differences in these genetic variants between the populations and ethnicities need consideration.

### 3.7. Polygenic Risk Scores

Due to the limitation of a single genetic variant to explain the NAFLD risk, developing a polygenic risk score (PRS) is favorable. In a study to determine the causal relationship between steatosis and the development of liver damage, a PRS model was developed based on the risk alleles of four genes (*PNPLA3*, *TM6SF2*, *GCKR,* and *MBOAT7*) that were associated with hepatic steatosis [186]. Each variant association with liver damage was proportional to its association with steatosis. Notably, the PRS model (steatosis risk alleles) association with liver damage was stronger than the conventional risk association via histological steatosis assessments [186]. These findings indicate that genetic risk alleles may reflect the actual long-term effects of steatosis in contributing to liver damage, and the histological assessment of steatosis may undermine the effects of steatosis on liver damage. Other studies also used similar PRS model and improved the diagnosis of steatosis in adults [187] and children [188] and HCC risk [189]. A PRS model based on three common variants of *PNPLA3*, *TM6SF2*, and *MBOAT7* genes was also associated with a higher risk of liver damage [47] and HCC risk [190].

In the Asian populations, a study of Korean NAFLD individuals showed that the PRS model (*PNPLA3*, *TM6SF2*, IR, liver enzymes, C-reactive protein, and diabetes status) predicted NASH risk with the area under the curve (AUROC) of 0.835 and 0.809 in NAFLD individuals with and without diabetes, respectively [115]. For Japanese individuals, a PRS model based on the (*PNPLA3*, *GCKR*, and *GATAD2A*) was associated with the NAFLD risk, and the risk was higher with the accumulation of the risk alleles [74]. Two Chinese studies showed that both *PNPLA3* and *TM6SF2* risk alleles confer a higher risk of having NAFLD in an additive manner [86,191].

Other studies reported the PRS model with the addition of the protective variant *HSD17B13* gene and NAFLD risk. One study investigated the potential of a PRS model based on three variants (*PNPLA3*, *TM6SF2*, and *HSD17B13*) to predict cirrhosis. The PRS model was able to predict the cirrhosis risk by 12-fold compared to the general population [192]. A recent multiethnic study of Americans (Caucasian, African, and Hispanic Americans) investigated the potential of multiple candidate genes to predict HCC risk among NAFLD individuals [193]. In this study, two PRS models were developed, namely the PRS-HFC (a model calculated by the sum of the effects of *PNPLA3*-*TM6SF2*-*MBOAT7*-*GCKR* risk alleles) and the PRS-5 (a PRS-HFC model that also incorporated the protective allele of *HSD17B13*). Both PRS models predicted the HCC risk better than a single gene risk score in NAFLD individuals (three-fold higher). Moreover, the prediction of HCC risk was independent of severe fibrosis, particularly in younger individuals and individuals with T2D [193]. This finding is essential, as many individuals are often not screened for HCC risk if no evidence of fibrosis exists. Since PRSs models are easily measured based on a single blood test and often adjusted to possible environmental factors, predicting the future development of NAFLD complications in clinical settings is possible.

## 4. Genetic Diversity in NAFLD Risk

Since NAFLD is a multi-factorial disease, there is a considerable contribution of genetics in modulating the individual or ethnic variations for the disease severity and risk of mortality associated with NAFLD.

### 4.1. Genetic Diversity of PNPLA3 I148M

Among those above genetic variants, the *PNPLA3* I148M is the most replicated variant in various populations (Table 1). Most studies showed similar predisposition effects on NAFLD, in which the individuals carrying the G allele have a greater risk of having steatosis and NAFLD. However, the prevalence of NAFLD and *PNPLA3* I148M variant frequency differs among populations.

The frequency of the I148M variant was highest in the Hispanic (0.49) compared to European (0.23) and African (0.17) Americans. Consistently, the observed levels of hepatic lipid contents in those NAFLD individuals also have a similar trend, with Hispanics having the highest hepatic lipid content, whereas African Americans have the lowest [34]. Thus, this variant I148M may partly explain the hepatic steatosis variability between the different ethnicities. Moreover, another *PNPLA3* variant, rs6006460 (S453I), was reported to associate with protective effects on NAFLD risk in African Americans. This protective effect was consistent with the higher frequency of this S453I variant in Africans (~10%) compared to other ethnicities (<1%) [34], confirming the genetic contribution to a lower risk of NAFLD among African individuals. Interestingly, the I148M variant is also associated with a “lean” NAFLD phenotype from Asia populations [5,63], suggesting a more specific relationship between the genetic variant and the subtypes of NAFLD geographically. However, a multiethnic Malaysian study showed that the I148M variant was associated with NAFLD risk, although the association was similar among the Malays, Chinese, and Indian ethnicities [55]. This discrepancy may be due to the small sample size for each ethnicity, which could undermine the statistical analysis. Furthermore, all three Malaysian ethnicities still belong to the Asian, and thus would have been different from the comparisons of Hispanics, Americans, Europeans, and Africans in other studies.

Another perspective is that the I148M variant could have sexual dimorphism on NAFLD risk or susceptibility. A meta-analysis of 16 studies reported a negative relationship between the male gender and the I148M variant on hepatic lipid content [122], and the effect of this variant on NAFLD risk was higher in women [39,122]. In contrast, the overall prevalence of NAFLD is higher in men globally [194], with countries such as the USA, Spain, and China having more men develop NAFLD [195]. However, other studies in Sri Lanka and Thailand showed an increase in NAFLD prevalence in women [195], and the overall median age of women who develop NAFLD is greater than that in men [196]. Consistent with this, the overall NAFLD prevalence in men becomes minimal in individuals over 50 years of age [194], implying the menopausal effects on the risk for NAFLD in women. Since estrogen is an essential hormone in lipid regulation and metabolism [197], the female gender could play a role in modulating the effects of I148M on NAFLD risk. However, this gender-specific effect requires further validation as most previous studies of the I148M variant did not perform gender-specific analysis.

### 4.2. Genetic Diversity of Other NAFLD Variants

The ethnic differences in other NAFLD variants are also evident. One such variant is the rs641738 in the *MBOAT7*, which was associated with NAFLD risk in Italians and Europeans, but not Asians (Table 1) [198]. In contrast, the association between the *APOC3* rs2854116 [T-455C] variant with NAFLD risk and hepatic lipid content was observed in Indian populations [162,163,199] but not in Western populations [40,53]. Similarly, some of the common NAFLD variants associations identified in Western populations did not replicate in some Asian populations. For example, Iranian studies showed that the *PPARGC1A* G482S [152], not the *GCKR* rs780094 variant [97], is associated with the NAFLD risk. On the other hand, the *TM6SF2* E167K variant did not associate with NAFLD risk in the Japanese [91] and Brazilian [54] populations. Interestingly, the *PNPLA3* I148M variant did not associate with the NAFLD risk in Filipino, despite this variant being higher in frequency in cases [73]. However, the sample size is small for an accurate conclusion.

In Malaysia’s multiethnic studies, three SNPs (rs3772627, rs2276736, and rs3772630) in the *AGTR1* gene have no association with the NAFLD risk in all patients. Though, a sub-analysis of the ethnicity showed that in the Indian ethnic group, these *ATGR1* SNPs have a significant protective effect on NAFLD risk, and no such association was observed in the Malays and Chinese Malaysians [164]. A similar subethnic analysis for the association with NAFLD and hepatic lipid content was done for the Indian population. The *PNPLA3* I149M variant was significantly associated with NAFLD risk in North Indians, whereas the *TM6SF2* E167K variant was associated with NAFLD in South Indians [90]. These differences may be due to the recent findings of the genomic profile of the North Indians are similar to Europeans and the South Indians to Asians [200]; thus, they may have a different genetic susceptibility.

### 4.3. Discrepancies between the Same Populations or Ethnicities

There are also discrepancies in the genetic findings of NAFLD within the same population or ethnicities. For example, a Chinese study of 112 adult NAFLD patients and 120-matched controls showed that the *PNPLA3* I148M variant increases the susceptibility of having NAFLD though no association was observed in steatosis grade [58]. However, another study of Chinese NAFLD individuals (203 adult patients with 202 matched controls) reported that the I148M variant was associated with the steatosis grade and other parameters such as BMI and plasma liver enzyme level [41]. Both of these studies have similar numbers of individuals; however, the method to confirm the steatosis was different. The latter study used an ultrasonography tool instead of a liver biopsy to confirm the steatosis [41]. Using ultrasound to diagnose steatosis is less accurate for individuals with lower-grade steatosis than higher-grade steatosis [201]. Thus, the diagnosis to exclude the presence of lower-grade steatosis may be compromised; therefore, the usage of ultrasound may contribute to the discrepancies in the findings.

Other discrepancies were also observed in studies of Japanese NAFLD individuals. The first Japanese study of 253 adult NAFLD individuals and 578 controls showed that the I148M variant increases the susceptibility of having NAFLD and fibrosis. However, no association was observed in steatosis grade [60]. Another study of 392 Japanese NAFLD individuals and 934 controls showed an association between the I148M variant with steatosis grade and fibrosis [48]. Both studies were done by the same group of researchers and used the same methods to diagnose and classify the clinical assessments, except that the latter study performed GWAS analysis, whereas the previous study used a candidate SNP approach. Significant evidence showed that the candidate-gene approach has shortcomings, including selection and publication bias and poor replication [202]. Therefore, this may explain the differences seen between these two Japanese studies.

Another NAFLD variant, the *GCKR* P446L variant, was reported with different findings among the Chinese populations. Two studies showed no association between the P446L variant and the susceptibility of NAFLD [98,102], whereas two studies reported that the P446L variant increases the susceptibility of having NAFLD [99,100]. The differences between these studies are the number of individuals included and the presence of other metabolic syndromes or diseases in the patients. Two studies that reported no association between the P446L variant relationship and NAFLD susceptibility also investigated the effects of metabolic syndrome [102] and coronary artery disease [98]. Thus, it may undermine the statistical analyses performed to determine the relationship between the P446L variant and NAFLD risk.

### 4.4. Challenges in NAFLD Genetic Diversity Research

Most of the NAFLD genetic polymorphism reported consistent effects between different populations. However, some discrepancies may be attributed to the ethnic-specific effect or the unknown genetic factors that have not been discovered yet. Furthermore, most previous findings are from the Western and East Asian populations and thus may undermine the role of genetics in NAFLD, especially in minorities. The Asia region has the second highest overall prevalence of NAFLD at 25% [203], and focusing on South East Asia, the overall prevalence was 42% [204]. Among the South East Asia countries, Indonesia ranks at the top with a NAFLD prevalence of 51%, followed by Singapore at 40% and Malaysia at 39% [204]. However, despite the high NAFLD prevalence, the genetic information from these South East Asians and the underrepresented Malay ethnicity was limited. Similarly, genetic polymorphism data is scarce for the Middle East region. More publications and reports from these two regions are needed to understand the effects of genetics in conferring the NAFLD risk. Moreover, genetics and environmental (geographic and socioeconomic) factors interact with each other to confer the NAFLD risk and resulting different subethnic effects. For example, the NAFLD prevalence among American Hispanics differs based on the ancestry origin of the individuals. NAFLD prevalence was highest for Hispanic individuals of Mexican origin (33%) compared to Hispanics of Puerto Rican origin (18%) and Dominican origin (16%) [205]. This finding is important as it shows that sub-ethnic diversity exists; thus, the traditional grouping of race or ethnicity may undermine the genetic contribution to NAFLD risk.

The current review is limited due to the lack of data from underrepresented countries and populations. The comparisons for genetic diversity might be biased and limit the generalizability of these genetic variants’ roles in the development of NAFLD disease. Another factor is that the small sample sizes and low statistical powers of the Asian studies [206] may undermine the actual NAFLD risk associations; therefore, their risk may not differ from the Western populations. Notably, these underpowered studies are often included in most meta-analysis studies despite there is a risk of publication bias. Underpowered studies tend to show extreme effects compared to large studies; though, this effect varied across meta-analyses [207]. In some meta-analyses with two or more large studies dominating the analysis, the underpowered studies have a minimal effect. Removing the underpowered studies will affect the precision if all studies have a similar sample size [207], thus performing the meta-analysis with a small sample size studies needs great cautions. Since sample size remains a significant issue in Asian studies, additional genetic information is vital to discover the unknown common and rare variants that could explain the individual risks and enhance understanding of NAFLD biology among Asians. Future studies could employ more in-depth genome sequencing (whole-exome sequencing or whole-genome sequencing) per individual to discover the low-frequency variants and CNVs [208]. This additional information from sequencing combined with GWAS is proven to work, as seen in the discovery of the new T2D variants that were otherwise missing from the GWAS studies alone [209]. Moreover, the extensive or deep phenotyping of the individuals could improve the analysis to detect new associations and heritability between the traits [210]. Participation in a large-scale international collaborative project will also allow for resources and knowledge transfer between the countries or populations and, thus, could improve the cohort sample size and diversity.

## 5. Conclusions

NAFLD prevalence and risk differ across populations. Genetic polymorphisms significantly influence the risk of having NAFLD and disease progression. However, most of the reported genetic studies are from Western populations and some from East Asians. There is a significant gap in the genetic information from other parts of Asia and minority ethnicities. The missing information is needed to address the ethnic-specific effects that could be used to tailor specific diagnostic tools and management programs. Future research is needed to address the genetic diversity of NAFLD and improve the understanding of the NAFLD disease development and progression.

## Figures and Tables

**Table 1:** Summary of the commonly reported single nucleotide polymorphism (SNP) associated with NAFLD.

Gene	SNP	Population Study	SNP Effects	Reference(s)
*PNPLA3*	rs738409 C > G (I148M)	Caucasians, African, Japanese, Latinos, Native Hawaiians, and European Americans, Amish, Family Heart Study, Framingham Heart Study, Finnish, Chinese, Taiwanese	Associated with NAFLD risk and steatosis	[34,35,36,37,38,39,40,41,42,43,44,45]
		German, Japanese	Associated with NAFLD risk, steatosis, and fibrosis	[46,47,48]
		Italian	Associated with NAFLD risk, steatosis, fibrosis, and cirrhosis	[49,50,51,52]
		Belgians	Associated with NAFLD and NASH risk	[53]
		Brazilian, Malaysians (Malays, Chinese, and Indians)	Associated with NAFLD and NASH risk, but not steatosis	[54,55]
		Turkish	Associated with NAFLD and NASH risk, and fibrosis	[56,57]
		Chinese	Associated with NAFLD risk but not steatosis	[58]
		Chinese	Associated with NAFLD risk in lean individuals	[59]
		Japanese	Associated with NAFLD risk and fibrosis, but not steatosis	[60,61]
		Korean	Associated with NAFLD risk and fibrosis	[62,63]
		Indian, Sri Lankans, Bangladeshi, Iranian, Singaporean (Chinese)	Associated with NAFLD risk	[64,65,66,67,68,69,70,71]
		Indian	Associated with NAFLD and NASH risk, and steatosis	[72]
		Filipinos	No association with NAFLD risk	[73]
	rs2896019 T > G	Japanese	Associated with NAFLD risk	[74]
	rs6006460 G > T (S453I)	African American	Associated with lower hepatic fat content	[34]
*TM6SF2*	rs58542926 G > A (E167K)	Caucasian, African, Hispanic, and European Americans, Swedish, European, Finnish, Polish, and Italian	Associated with NAFLD risk and steatosis	[49,52,75,76,77,78,79,80,81,82]
		German	Associated with NAFLD risk and steatosis but not fibrosis	[47]
		Caucasians, African, and Hispanic Americans, Chinese	Associated with NAFLD risk, steatosis, and fibrosis	[43,83,84,85,86,87]
		Italian, Finnish, and Swedish	Associated with NASH progression	[88]
		Korean	Associated with NASH and fibrosis	[89]
		Indian, Argentinian	Associated with NAFLD risk	[66,77,90]
		Brazilian, Japanese, Singaporean (Chinese)	No association with NAFLD	[54,71,91]
*GCKR*	rs780094 C > T	Amish, Family Heart Study, and Framingham Heart Study, Caucasian, African, and Mexican Americans	Associated with NAFLD risk and steatosis	[39,92]
		German	Associated with NAFLD and fibrosis severity	[46]
		Italian	Associated with liver fibrosis severity	[93]
		Taiwanese, Uygur	Associated with NAFLD risk	[94,95]
		Malaysians (Malays, Chinese, and Indians)	Associated with NAFLD, NASH, and fibrosis	[96]
		Iranian, Chinese, Singaporean (Chinese)	No association with NAFLD	[71,97,98]
	rs1260326 C > T (P446L)	Italian, Japanese, Uygur, and Chinese	Associated with NAFLD risk	[49,74,95,99,100]
		Finnish	Associated with steatosis	[101]
		Malaysians (Malays, Chinese, and Indians)	Associated with NAFLD, NASH, and fibrosis	[96]
		Chinese	No association with NAFLD	[98,102]
*MBOAT7*	rs641738 C > T	Dallas Heart Study and Europeans, Italian	Associated with NAFLD risk and steatosis	[49,52,103]
		Finnish	Associated with NASH and fibrosis	[104]
		German	Associated with fibrosis only	[47]
		Eastern European	No association with fibrosis or cirrhosis	[105]
		Americans, Italian, Argentinian, Chinese, Taiwanese, and Korean	No association with NAFLD risk	[37,89,102,106,107,108]
*HSD17B13*	rs72613567 T > TA	Caucasian Americans	Associated with steatosis and lower risk of inflammation and NASH	[109,110]
		European, Finnish	Associated with lower NASH and fibrosis risk	[111,112]
		Argentinian, Japanese	Associated with lower NASH risk	[113,114]
		Korean	Associated with lower NAFLD risk	[115]

Abbreviation: GCKR: glucokinase regulatory protein; HSD17B13: hydroxysteroid 17β-dehydrogenase; MBOAT7: membrane-bound O-acyltransferase domain-containing 7; NAFLD: nonalcoholic fatty liver disease; NASH: nonalcoholic steatohepatitis; PNPLA3:patatin-like phospholipase domain-containing 3; TM6SF2: transmembrane 6 superfamily member 2.

## Data Availability

Not applicable.
